# Rethinking childhood ependymoma: a retrospective, multi-center analysis reveals poor long-term overall survival

**DOI:** 10.1007/s11060-017-2568-8

**Published:** 2017-07-21

**Authors:** Amanda E. Marinoff, Clement Ma, Dongjing Guo, Matija Snuderl, Karen D. Wright, Peter E. Manley, Hasan Al-Sayegh, Claire E. Sinai, Nicole J. Ullrich, Karen Marcus, Daphne Haas-Kogan, Liliana Goumnerova, Wendy B. London, Mark W. Kieran, Susan N. Chi, Jason Fangusaro, Pratiti Bandopadhayay

**Affiliations:** 1 Dana-Farber Cancer Institute and Boston Children’s Hospital, Boston, MA USA; 2 Pediatric Neuro-Oncology Program, Dana-Farber/Boston Children’s Cancer and Blood Disorders Center, Dana-Farber Cancer Institute and Boston Children’s Hospital and Harvard Medical School, 450 Brookline Avenue, Boston, MA 02215 USA; 3 Department of Neurosurgery, Dana-Farber Cancer Institute and Boston Children’s Hospital, Boston, 02215 MA USA; 4 Department of Neurology, Dana-Farber Cancer Institute and Boston Children’s Hospital, Boston, 02215 MA USA; 5 Departments of Pathology and Neurology, New York University Langone Medical Center and Medical School Pathology, New York, NY USA; 6 Pediatric Neuro-Oncology Program, Ann & Robert H. Lurie Children’s Hospital of Chicago, Chicago, IL USA

**Keywords:** Ependymoma, Survival, Resection, Grade, Outcome

## Abstract

Ependymoma is the third most common brain tumor in children, but there is a paucity of large studies with more than 10 years of follow-up examining the long-term survival and recurrence patterns of this disease. We conducted a retrospective chart review of 103 pediatric patients with WHO Grades II/III intracranial ependymoma, who were treated at Dana-Farber/Boston Children’s Cancer and Blood Disorders Center and Chicago’s Ann & Robert H. Lurie Children’s Hospital between 1985 and 2008, and an additional 360 ependymoma patients identified from the Surveillance Epidemiology and End Results (SEER) database. For the institutional cohort, we evaluated clinical and histopathological prognostic factors of overall survival (OS) and progression-free survival (PFS) using the log-rank test, and univariate and multivariate Cox proportional-hazards models. Overall survival rates were compared to those of the SEER cohort. Median follow-up time was 11 years. Ten-year OS and PFS were 50 ± 5% and 29 ± 5%, respectively. Findings were validated in the independent SEER cohort, with 10-year OS rates of 52 ± 3%. GTR and grade II pathology were associated with significantly improved OS. However, GTR was not curative for all children. Ten-year OS for patients treated with a GTR was 61 ± 7% and PFS was 36 ± 6%. Pathological examination confirmed most recurrent tumors to be ependymoma, and 74% occurred at the primary tumor site. Current treatment paradigms are not sufficient to provide long-term cure for children with ependymoma. Our findings highlight the urgent need to develop novel treatment approaches for this devastating disease.

**Electronic supplementary material:**

The online version of this article (doi:10.1007/s11060-017-2568-8) contains supplementary material, which is available to authorized users.

## Introduction

Ependymoma is the third most common brain tumor in children, accounting for 6–10% of all intracranial tumors [1]. In children, approximately 90% of ependymomas are intracranial, with about two-thirds arising within the posterior fossa [2]. Ependymomas are histologically classified as grade I (subependymomas and myxopapillary ependymoma), grade II (classic ependymomas), and grade III (anaplastic ependymomas) tumors; however histological criteria have poor predictive value [3]. Standard therapy typically consists of maximal surgical resection, followed by post-operative radiation therapy directed at the site of the primary tumor [4]. While there is a long history of subclassifying ependymoma by histology, currently, there exists little treatment stratification for ependymoma, and the long-term prognosis for patients with this disease remains poorly understood.

In contradistinction to most other primary brain tumors, clinical, pathologic, and radiologic factors that influence outcomes for patients with ependymoma have not been well defined [5]. Although complete resection has long been shown to predict better outcomes, research has not yielded consistent findings with regard to the prognostic significance of other common factors such as age, tumor location and tumor grade [6–11]. Recently, however, large-scale genomic and epigenomic studies have revealed distinct molecular subgroups associated with differential prognoses [12–27].

Compounding these obstacles is the paucity of reports that have included large single- or multi-institutional pediatric populations with long-term follow-up. Most previous studies report 3- and 5-year survival outcomes [5, 28] and those that report longer-term outcomes have included relatively small numbers of patients and/or follow-up time less than 10 years [21, 29–34].

We performed detailed outcome analyses with extended follow-up on 113 pediatric patients with intracranial ependymoma who were treated at two pediatric institutions between 1985 and 2008. In addition, 360 ependymoma patients identified from the Surveillance Epidemiology and End Results (SEER) database were included as a reference population.

### Materials and methods

This HIPAA-compliant study was approved by the Institutional Review Boards of the Dana-Farber Cancer Institute (DFCI)/ Boston Children’s Hospital (BCH) and Ann & Robert H. Lurie Children’s Hospital of Chicago (LCH). Many of the patients reported from the LCH cohort have been previously reported in a study which evaluated the utility of cerebrospinal fluid (CSF) examination in ependymoma patients [35].

### Patient cohort

We performed a retrospective review of 463 patients ≤18 years of age at diagnosis with WHO Grade II and Grade III intracranial ependymoma (as defined by the WHO modified criteria) [36], which included two independent cohorts: an institutional cohort and a validation cohort from the Surveillance, Epidemiology, and End Results (SEER) registry (1973–2013). The institutional cohort included 113 patients who were treated between 1985 and 2008 at DFCI/BCH (N = 52) or LCH (N = 61). Of those, 10 patients (4 from Boston; 6 from Chicago) were excluded due to presence of metastatic disease at diagnosis. The final institutional cohort thus included 103 patients (48 from Boston; 55 from Chicago).

For the SEER validation cohort, we extracted 1054 ependymoma patients ≤18 years of age at diagnosis from the November 2013, dataset by querying “ICCC site rec extended ICD 0 3/WHO 2008” with the term “ependymoma” [37]. In order to match our study’s inclusion criteria (WHO grade II and III ependymoma), we excluded 685 patients with grade I or unknown grade tumors. A total of 360 SEER patients were included in the analysis.

### Patient clinical histopathology and immunohistochemistry data

Patient records were abstracted to obtain information regarding date of birth, gender, date of diagnosis, disease site, extent of surgical resection, adjuvant therapy including radiation and/or chemotherapy, presence and site of recurrent disease, date of recurrence, date and disease status at last follow-up, including survival.

Surgical resections were graded as gross total resection (GTR) or subtotal resection (STR) by reviewing the post-operative MRI, or if not available, the post-operative report. A gross total resection was defined as absence of residual disease at the primary tumor site by post-operative MRI imaging in most cases and by intra-operative inspection in those without available imaging. Any residual tumor noted at the time of surgical resection was considered a subtotal resection. The follow-up duration for each patient was calculated. Disease status at follow-up was determined from radiology reports. Disease status was classified as no evidence for disease (NED), alive with disease, or death from disease.

Histopathological features, including architecture (classic/WHO Grade II or anaplastic/WHO Grade III ependymoma), presence of necrosis, vascular proliferation, and mitotic index were determined by a senior neuropathologist for the 48 tumor samples available at DFCI/BCH (MS). Immunohistochemistry performed in each of these cases enabled analysis of the MIB-1 labeling index, topoisomerase-II alpha (topo-IIα) expression, and expression of p53, Bcl-2, and cyclin D. Previous reports indicate that these markers may significantly predict survival outcomes in children with ependymoma [38]. To characterize MIB-1 and topoisomerase-II alpha expression, the fraction of immunolabeled tumor cell nuclei was expressed as a percentage (index). Patients were separated into low and high index groups using previously reported cutoffs (MIB-1: low index ≤20.5%; high index >20.5%; topo-IIα: low index ≤9.4%; high index >9.4%) [39]. The thresholds for both indices were less than one standard deviation above our mean proliferation rates. Histology at disease recurrence was determined subsequent to biopsy, surgical resection, or autopsy by review by a neuropathologist at DFCI/BCH or LCH following the WHO 2007 diagnostic criteria [36].

## Statistical methods

Overall survival (OS) was calculated from date of diagnosis to date of death, or until date of last follow-up if the patient was alive. Tumor recurrence was defined radiologically as greater than 25% growth of an existing lesion or development of disseminated disease. Progression-free survival (PFS) was calculated from date of diagnosis to recurrence or date of death, or until date of last follow-up if the patient was alive.

Fisher’s Exact Test was used to compare categorical factors between groups and Wilcoxon rank-sum test was used for continuous factors. Kaplan–Meier curves of OS and PFS were generated and log-rank tests were used to compare OS and PFS between demographic and clinical factors. We performed multivariate Cox proportional hazards regression to identify significant prognostic factors for OS and PFS; in each model, we started with all significant prognostic factors from the univariate analysis and performed backwards variable selection to reach the final multivariate model, and checked for evidence of non-proportional hazards. All analyses were performed using SAS version 9.4 (Cary, NC) and two-sided p values <0.05 were considered statistically significant.

## Results

### Demographic and clinical characteristics

The demographic and clinical characteristics of the institutional cohort are summarized in Table 1. Median age at diagnosis of these children was 3.6 years (range 0.6–18.2 years); 49 (48%) patients were male. Median follow-up time was 11 years (range 0.2–28 years). Twenty-five (24%) patients had supratentorial ependymomas and 78 (76%) had infratentorial tumors. Seventy-five (73%) patients had Grade II (non-anaplastic) ependymoma and 28 (27%) had Grade III (anaplastic) pathology. GTR was achieved in 64 (62%) of patients while 39 (38%) had a STR. Adjuvant treatment regimens following resection included radiation therapy only (41%), chemotherapy only (11%), radiation and chemotherapy (40%), or observation only (9%).

**Table 1:** Patient demographic and clinical characteristics by institution

Demographic and clinical characteristics	DFCI/BCH (n = 48)	LCH (n = 55)	All patients (n = 103)	p value*
Median (range)				
Age at diagnosis (years)	3.5 (0.6, 18.2)	3.7 (0.6, 16.4)	3.6 (0.6, 18.2)	0.9
Follow-up time in surviving patients (years)	13.9 (0.7, 27.6)	8.9 (0.17, 21.5)	11 (0.17, 27.6)	0.11
Frequency (%)				
Gender				
Male	25 (52)	24 (44)	49 (48)	0.4
Tumor location				
Infratentorial	36 (75)	42 (76)	78 (76)	1.0
Supratentorial	12 (25)	13 (24)	25 (24)	
Tumor grade				
II	35 (73)	40 (73)	75 (73)	1.0
III	13 (27)	15 (27)	28 (27)	
Site of recurrence				
Local only	19/34 (56)	32/35 (91)	51/69 (74)	0.005
Intracranial dissemination only	4/34 (12)	1/35 (3)	5/69 (7)	
Distant spine only	3/34 (9)	1/35 (3)	4/69 (6)	
Local + distant spine only	8/34 (24)	1/35 (3)	9/69 (13)	
Extent of resection				
GTR	28 (69)	36 (67)	64 (62)	0.5
STR	20 (31)	19 (33)	39 (38)	
Treatment				
XRT only	21 (43)	20/53 (38)	41/101 (41)	<0.0001
Chemo only	0 (0)	11/53 (21)	11/101 (11)	
Chemo + XRT	27 (56)	13/53 (26)	40/101 (40)	
Observation	0 (0)	9/53 (17)	9/101 (9)	

*Fisher’s exact test was used to test categorical factors and Wilcoxon rank sum test was used for continuous factors

When comparing patient characteristics between institutions, the only significant difference was in post-operative treatment regimens (p < 0.0001, Table 1); patients treated at LCH were more likely to be observed post-operatively (17 vs. 0%) or to receive chemotherapy only (21 vs. 0%), and less likely to be treated with combined radiation and chemotherapy (26 vs. 56%). All other patient characteristics, including age, gender, tumor location, tumor grade, and extent of resection, did not differ significantly across institutions (p > 0.05).

Histopathological characteristics for 48 patients treated at DFCI/BCH are summarized in Supplemental Table 2; these data were not available for LCH patients. Tumor architecture was classified as classic (Grade II) in 13 (27%) cases and anaplastic (Grade III) in 35 (73%) cases. Necrosis was present in 37 (77%) cases and vascular proliferation was observed in 29 (60%) cases. Immunohistochemistry revealed nuclear positivity of p53 protein in 28 (58%), Bcl-2 positivity in 42 (88%), and cyclin D positivity in 36 (75%) of cases. The median number of mitoses per HPF was 2.5 (range 0–27). The median MIB-1 LI was 15.4 (range 0.8–45); 17 (36%) had a MIB-1 LI ≥ 20.5. The median topo-IIα expression was 6.1% (0–31%); 14 (29%) had topo-II alpha expression ≥9.4%.

The SEER cohort included 360 ≤ 18 years, of which 206 (57%) were male. Available demographic data is shown in Supplemental Table 1. Eight-two (23%) had Grade II (classic) ependymoma and 281 (77%) had Grade III (anaplastic) pathology. 241 (67%) patients received adjuvant radiation therapy, 113 (31%) patients did not receive radiation, and in 7 (2%) cases, use of adjuvant radiation was unknown.

### Children with ependymoma exhibit poor long-term survival outcomes

**Fig. 1:**
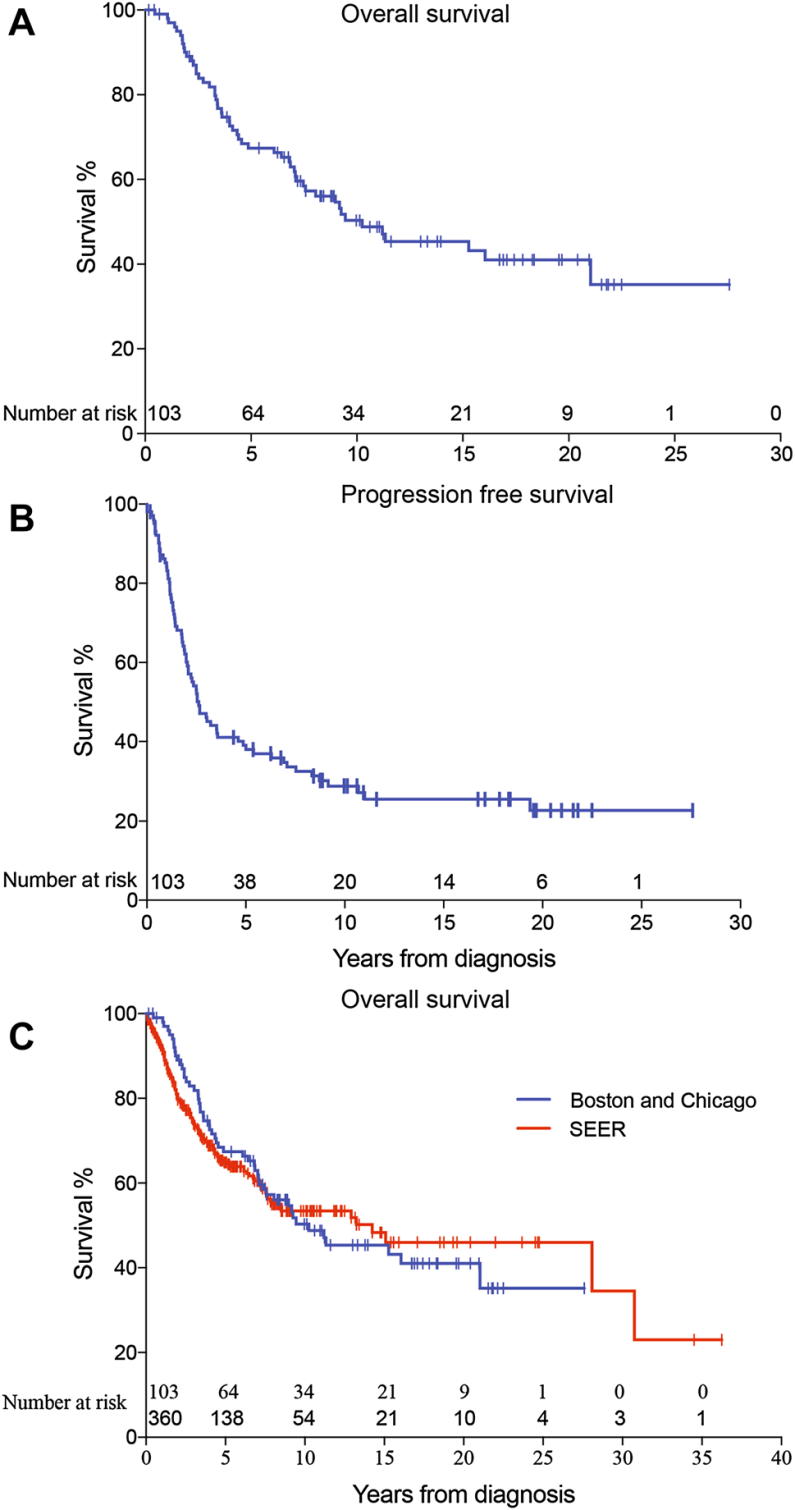
Kaplan–Meier curves of **a** overall survival (OS) and **b** progression-free survival (PFS) of the study cohort (n = 103) **c** comparison of OS of the study cohort (n = 103) and SEER cohort (n = 360)

Children with ependymoma in the institutional cohort exhibited 5-year OS and PFS rates of 67 ± 5% and 39 ± 5%, respectively (Fig. 1a, b). However, survival curves did not plateau after 5 years; 10 year OS and PFS were 50 ± 5% and 29 ± 5%, respectively. We did not observe significant differences in OS or PFS between institutions (Supplemental Fig. 1; log-rank p ≥ 0.6). Five-year OS was 63 ± 7% for DFCI/BCH patients and 71 ± 6% for LCH patients. We observed OS and PFS to continue to decline between 10 and 20 years.

The SEER cohort exhibited 5- and 10- year OS rates of 63 ± 3% and 52 ± 3%, respectively (Fig. 1c). Taken together, these data confirm that approximately half of all children diagnosed with ependymoma died of their disease by 10 years after diagnosis.

### Prognostic factors for survival outcomes

**Fig. 2:**
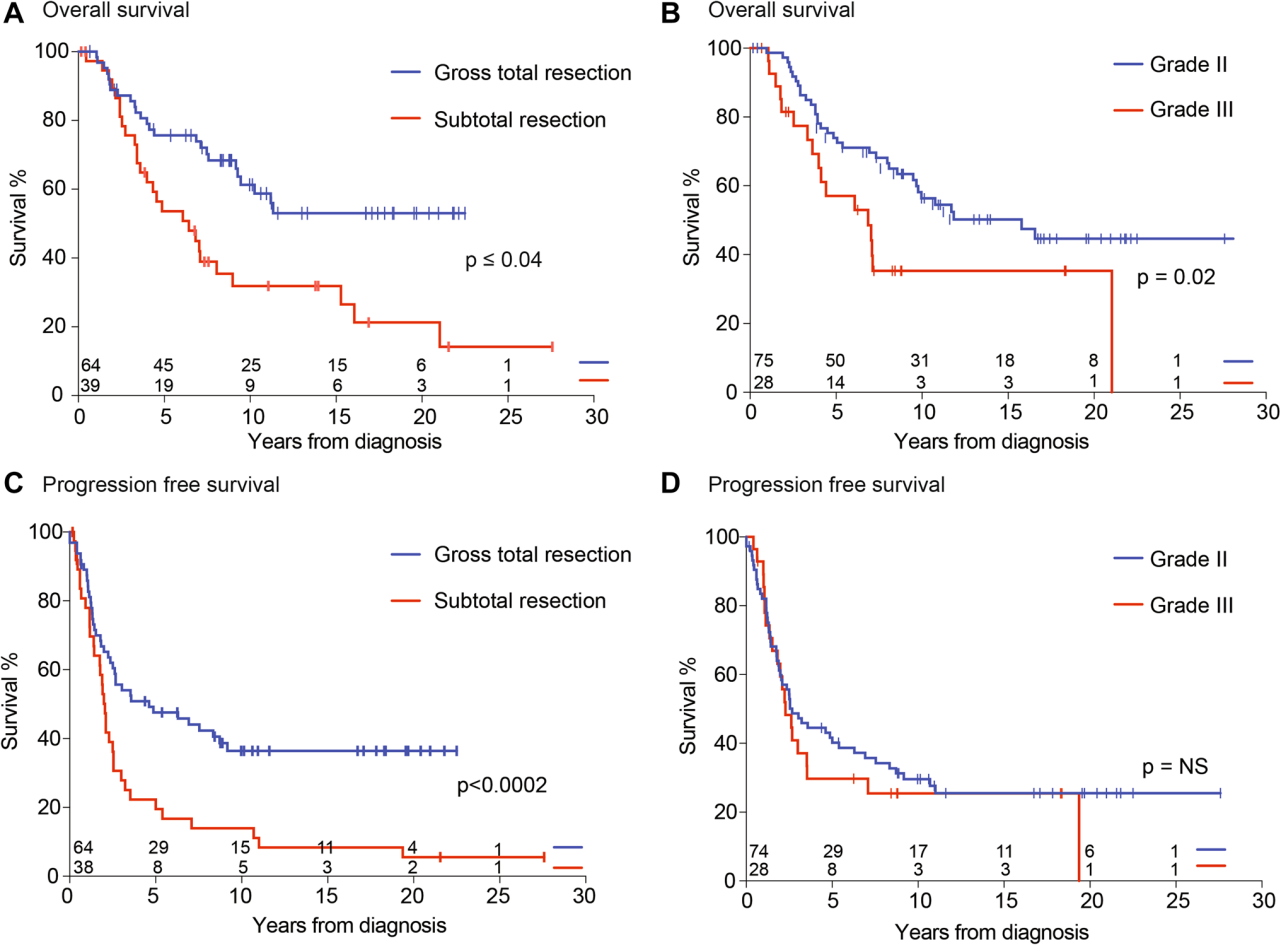
Kaplan–Meier curves of overall survival (OS) and progression free survival (PFS) by: (**a, c**) extent of resection and (**b, d**) tumor grade

Extent of resection, tumor grade and treatment type impacted overall survival. In univariate analyses of OS (Table 2), tumor grade, extent of resection, and treatment type were significantly associated with OS (log-rank p ≤ 0.03) (Fig. 2a, b). Grade II pathology was associated with significantly improved OS, compared to Grade III (anaplastic) pathology (5-year OS = 71 ± 5% vs. 57 ± 10%; p = 0.026). We confirmed extent of resection to be prognostic in our institutional cohort. GTR compared to STR was associated with significantly improved OS (5-year OS = 75 ± 5% vs. 54 ± 8%; p = 0.002; Fig. 2). Treatment type was significantly associated with OS (p < 0.0001); patients who received chemotherapy only or combined chemotherapy and radiation as part of their first treatment had significantly poorer OS (51 ± 8%) than patients treated with all other modalities.

**Table 2:** Univariate prognostic factors of overall survival and progression free survival

Demographic and clinical factors (n = 103)		Frequency (%)	5-year OS ± SE	10-year OS ± SE	OS p value*	5-year PFS ± SE	10-year PFS ± SE	PFS p value*
Institution	DFCI/BCH	48 (47)	63 ± 7	48 ± 8	0.6	43 ± 7	30 ± 7	0.8
	LCH	55 (53)	71 ± 6	52 ± 9		36 ± 7	27 ± 7	
Age at diagnosis	<3 years old	43 (42)	73 ± 7	57 ± 8	0.5	40 ± 7	32 ± 7	0.9
	≥3 years old	60 (58)	63 ± 6	46 ± 7		39 ± 6	27 ± 6	
Gender	Male	49 (48)	68 ± 8	47 ± 8	0.6	36 ± 7	25 ± 6	0.3
	Female	54 (52)	67 ± 7	54 ± 7		41 ± 7	33 ± 7	
Tumor location	Infratentorial	78 (76)	65 ± 6	51 ± 6	0.7	41 ± 6	32 ± 5	0.5
	Supratentorial	25 (24)	74 ± 9	45 ± 12		34 ± 10	19 ± 8	
Tumor grade	II	75 (73)	71 ± 5	56 ± 7	0.026	42 ± 6	30 ± 6	0.5
	III	28 (27)	57 ± 10	35 ± 16		30 ± 9	25 ± 8	
Site of recurrence^a^	Local only	51/69 (74)	60 ± 7	39 ± 7	0.2	14 ± 5	2 ± 2	0.7
	Intracranial dissemination only	5/69 (7)	75 ± 22	50 ± 25		40 ± 22	20 ± 18	
	Distant spine only	4/69 (6)	50 ± 25	50 ± 25		25 ± 22	25 ± 22	
	Local + Distant spine	9/69 (13)	44 ± 17	11 ± 10		22 ± 14	0	
Extent of resection	GTR	64	75 ± 5	61 ± 7	0.002	48 ± 6	36 ± 6	0.0022
	STR	39	54 ± 8	32 ± 8		24 ± 7	16 ± 6	
Treatment	XRT only	41/101	79 ± 7	73 ± 7	<0.0001	50 ± 8	42 ± 8	0.0016
	Chemo only	11/101	73 ± 13	45 ± 15		27 ± 13	18 ± 12	
	Chemo + XRT	40/101	51 ± 8	26 ± 8		26 ± 7	14 ± 6	
	Observation	9/101	88 ± 12	88 ± 12		63 ± 17	63 ± 17	

Histopathologic features (n = 48)		Frequency (%)	5-year OS ± SE	10-year OS ± SE	OS p value*	5-year PFS ± SE	10-year PFS ± SE	PFS p value*
Architecture	Classic (WHO Grade II)	13	85 ± 10	67 ± 14	0.2	62 ± 13	44 ± 14	0.4
	Anaplastic (WHO grade III)	35	49 ± 11	38 ± 11		32 ± 10	27 ± 9	
Necrosis	No	11	82 ± 12	61 ± 15	0.1	36 ± 15	24 ± 14	0.8
	Yes	37	57 ± 8	44 ± 9		45 ± 8	32 ± 8	
Vascular proliferation	No	19	72 ± 11	61 ± 12	0.18	50 ± 12	39 ± 12	0.4
	Yes	29	57 ± 9	38 ± 10		38 ± 9	25 ± 9	
P53 status	Negative	20	74 ± 10	57 ± 12	0.5	55 ± 11	33 ± 11	0.7
	Positive	28	54 ± 10	41 ± 10		34 ± 9	29 ± 9	
Blc-2 status	Negative	6	100	50 ± 25	0.3	67 ± 19	44 ± 22	0.08
	Positive	42	58 ± 8	47 ± 8		39 ± 8	28 ± 7	
MIB-1 LI	<20.5	31	74 ± 8	49 ± 10	0.9	52 ± 9	32 ± 9	0.8
	≥20.5	17	44 ± 12	44 ± 12		25 ± 11	25 ± 11	
Topo-II alpha expression	<9.4	34	75 ± 8	54 ± 9	0.052	49 ± 9	32 ± 8	0.2
	≥9.4	14	36 ± 13	36 ± 13		29 ± 12	29 ± 12	
Mitotic index	≤10	40	67 ± 8	49 ± 8	0.3	45 ± 8	31 ± 8	0.4
	>10	8	43 ± 19	43 ± 19		29 ± 17	29 ± 17	
Cyclin D expression	Negative	12	72 ± 14	48 ± 17	0.5	58 ± 14	29 ± 14	0.4
	Positive	36	60 ± 8	47 ± 8		37 ± 8	31 ± 8	

^a^Restricted to patients who relapsed

*p value of log rank test

In the multivariate Cox proportional hazards model (Table 3), only tumor grade and treatment type remained significant (p < 0.03) after backwards selection; the proportional hazards assumption was upheld. In univariate analyses of PFS (Table 2), extent of resection and treatment type were significantly associated with PFS (p < 0.003) (Fig. 2c); tumor grade did not confer prognostic significance (Fig. 2d). In the multivariate model, only adjuvant treatment type remained significant after backward selection.

**Table 3:** Multivariate Cox proportional hazards model for overall survival (n = 101)

Predictive factor		HR (95% CI)	p value
Tumor grade	II	(Reference)	
	III	1.9 (1.1–3.5)	0.03
Treatment	Chemo + XRT	(Reference)	
	Chemo only	0.7 (0.3–1.6)	0.41
	Observation only	0.1 (0–0.7)	0.025
	XRT only	0.3 (0.1–0.5)	0.0001

Although GTR was significantly associated with improved survival, it was not curative for all children. While 75% (±5%) of all children who underwent a GTR were survivors at 5 years past diagnosis, we observed late recurrences and deaths. By 10 years, OS for patients treated with a GTR was 61 ± 7% and PFS was 36 ± 6%. Forty-nine of 64 patients (76%) who underwent a GTR had also received adjuvant radiation therapy at diagnosis.

### Pathology confirms relapses are due to recurrent ependymoma

Pathological examination confirmed recurrent tumors to be ependymoma. Forty-two of the 69 patients with recurrent disease on imaging (62.7%) underwent a surgical procedure at first recurrence that enabled pathological confirmation of the recurrent tumor. Among these 42 recurrent tumors, 39 tumors (93%) were consistent with ependymoma. In five patients (20%) with an initial diagnosis of WHO Grade II ependymoma, pathology at last recurrence revealed a Grade III ependymoma. Of the remaining four tumors, two were reported as high-grade diffuse gliomas and one a meningioma. These findings confirm that nearly all relapses were due to recurrent ependymoma, rather than radiation-associated secondary malignant gliomas.

### The majority of relapses occur at the primary tumor site, independent of prior use of cranial irradiation

Despite therapy to achieve local disease control, we observed the majority of relapses to occur at the local tumor site. Among the 69 patients who displayed evidence of disease recurrence and could be evaluated for site of relapse, 51 (74%) had an isolated local relapse, 9 (13%) had concurrent local relapse with metastatic disease to the spine, and 4 (6%) had isolated spine metastases, and 5 (7%) had intracranial dissemination at first recurrence. In one case, the site of relapse was unknown. Among the 50 patients with isolated local disease recurrence, 40 (58%) had been treated with radiation therapy. Site of recurrence was not significantly influenced by treatment type: 40/58 (69%) patients who received radiation therapy experienced a local recurrence compared to 11/11 (100%) patients who did not (p = 0.55). Of those who did not receive radiation 7/11 (64%) underwent a GTR.

These data suggest that control at the primary site remains a major positive predictor of long-term survival. Moreover, our data suggest that current strategies for local disease control with gross total resection when feasible, followed by focal radiation therapy to the primary tumor site, are not sufficient to prevent late-occurring relapses and deaths.

## Discussion

Our series, the largest multi-institutional series of children with ependymoma with more than 10 years of median follow-up, demonstrates that children with ependymoma face poor long-term survival, even after gross total resection and treatment with adjuvant radiotherapy and/or chemotherapy. These data suggest that current treatment paradigms are not curative for the majority of children and that novel therapeutic strategies are required for this disease.

We have shown that long-term outcomes for children with ependymomas are dismal. We found that 10-year OS is 50 ± 5% and PFS is 29 ± 5%. Importantly, these outcomes are reproduced in two independent academic centers and validated with SEER data. Our experience, with 103 patients and a median of 11 years of follow-up time, is in agreement with prior studies with smaller patient cohorts and/or shorter follow-up periods [5, 29–33, 40]. While Merchant et al. found 5-year OS of 85% and EFS of 74% in a prospective study of 153 patients, the median follow-up period for this study was only 5.3 years, with only 14 patients alive at 10 years, compared to 38 patients in this study [32]. In our study, we found that half of all children with ependymoma continue to relapse and die of their disease after more than a decade from diagnosis. These data have potential implications for altering current treatment strategies, as well as the approach to counseling patients and families on prognosis for ependymoma at initial diagnosis.

Importantly, our study reveals that GTR is not curative in many children. We found that while 5-year OS is 75 ± 5% in children with completely resected tumors, 10-year OS drops to 61 ± 7%. It is well established that GTR of ependymoma is the most important clinical predictor of superior PFS and OS [32]. We observed that while GTR was associated with improved OS and PFS compared to STR, it is often insufficient to prevent late-occurring relapses and deaths. Thus, even when GTR is possible, the natural history for many patients treated with the current standard of care is recurrence and death from their disease.

Our data suggest that traditional clinical and histopathological variables do not provide a sufficient basis for risk stratification of children with ependymoma, and that molecular data are needed to inform our understanding of patient prognosis. Previous studies have not yielded consistent findings with regard to the prognostic significance of tumor grade [1, 4, 5, 7–9, 31, 40]. This heterogeneity across studies may reflect the lack of uniform criteria for histopathological classification of ependymoma, as well as the use of discrete categories to describe a disease that may be better understood along a pathological spectrum and more meaningfully defined according to molecular subtypes. In addition to the need to prospectively validate the robust molecular classification system proposed by Pajtler et al. [18], we expect that elucidating the role of additional molecular subgroups, copy number alterations, and epigenetic alterations will be instrumental to further understanding ependymoma biology and refining patient risk stratification. In particular, H3K27me3 immunostaining, which has recently been revealed as a promising biomarker in posterior fossa ependymomas [41], would be both valuable and viable to incorporate into future studies investigating outcomes and potential therapeutic targets.

We confirmed that the majority of recurrent tumors are histologically ependymomas. Although radiation can cause secondary malignancies, 93% of relapses in the institutional cohort were due to recurrent ependymoma, most frequently at the local tumor site, highlighting the failure of current treatment strategies to provide local control and long-term cure. In the SEER Cancer Statistics Review, 1975–2000, the cumulative incidence of a subsequent cancer developing among cancer survivors was 5.0% at 5 years and 8.4% at 10 years [42]. Given our median follow-up of 11 years, the risk of second malignancy in our cohort is comparable to the cumulative incidence of second malignancy among cancer survivors in that publication. It should be noted, however, that our analysis of the incidence of secondary malignancies was limited by the relatively small number of recurrent tumors available for pathologic review (n = 48), as well as limited information regarding the genetic background and cumulative radiation dose for each patient. It is also important to recognize that radiation-induced second primaries would be expected to rise with longer follow-up periods, with a high incidence of radiation-induced meningiomas arising after exceptionally long latency periods (>20 years after irradiation treatment). This further underscores the need for large, multi-institutional studies with long-term follow-up to assess patient outcomes and optimize surveillance protocols.

Treatment strategies for ependymoma are focused on local tumor control [4, 29, 30, 32–34]. However, we have observed children to exhibit late recurrences despite such treatment. Our data highlights the need to consider other therapeutic options for these children and provides a rationale for investigating the role of maintenance therapy after local control. The current COG trial ACNS 0831 assesses the efficacy of the addition of maintenance chemotherapy to standard local treatments [43]. Multi-center collaborative and molecularly informed trials are desperately required to assess the most effective maintenance therapies for children with this disease.

Limitations of this study include those inherent to a retrospective analysis of a rare tumor over a 20-year period including variability of imaging technologies across this time. In addition, there are limitations to the data obtainable from the SEER database. In particular, the quality of the data extracted is dependent on how the data are entered into the database, histology and radiology results have not been centrally reviewed, and there may be considerable variability in the grading of ependymomas due to changes in the WHO classification system across this time. A further limitation to this study is our analysis of supratentorial and infratentorial tumors as a single group. While combining the two molecularly distinct groups may have masked differences in outcome between biologically different tumors, we found no significant differences in OS or PFS between supratentorial and infratentorial tumors on univariate analysis; we thus opted to combine the groups in order to more robustly power our analysis.

While this study highlights the poor long-term outcomes for children with ependymoma, several questions remain to be answered. First, how does the molecular subtyping of ependymoma [12–27, 44–46] influence long-term outcomes, and does it allow long-term risk stratification? Second, what is the optimal adjuvant therapy for these children and how long should such treatment be considered? Third, what is the best strategy to implement targeted small-molecule inhibitors into upfront therapy? Further studies that incorporate long-term outcomes with molecular subtyping are needed to understand which children are at greatest risk for poor outcomes, and conversely, which children are likely to be long-term survivors who may be candidates for reduced intensity treatment regimens.

We have demonstrated that long-term survival for children with ependymoma is poor. Even children who receive the most optimal available treatment with GTR and adjuvant radiotherapy are at risk for relapse and death for more than a decade from diagnosis. Our findings highlight the urgent need to develop novel approaches for treatment that include adjuvant therapy for this devastating disease. Future research should focus on incorporating molecular subtyping to better understand differential patient prognosis and on reshaping treatment strategies to improve long-term outcomes.

## Electronic supplementary material

Below is the link to the electronic supplementary material.

Kaplan–Meier curves of overall survival (OS) and progression-free survival (PFS) by institution. Supplementary material 1 (PDF 41 KB)

Supplementary material 2 (PDF 86 KB)
