# Supplementary figures and images for: Rethinking childhood ependymoma: a retrospective, multi-center analysis reveals poor long-term overall survival
Source: J Neurooncol. 2017 Jul 21;135(1):201–11. doi: 10.1007/s11060-017-2568-8 (PMC5658456; doi:10.1007/s11060-017-2568-8)

## Overall Survival by Site

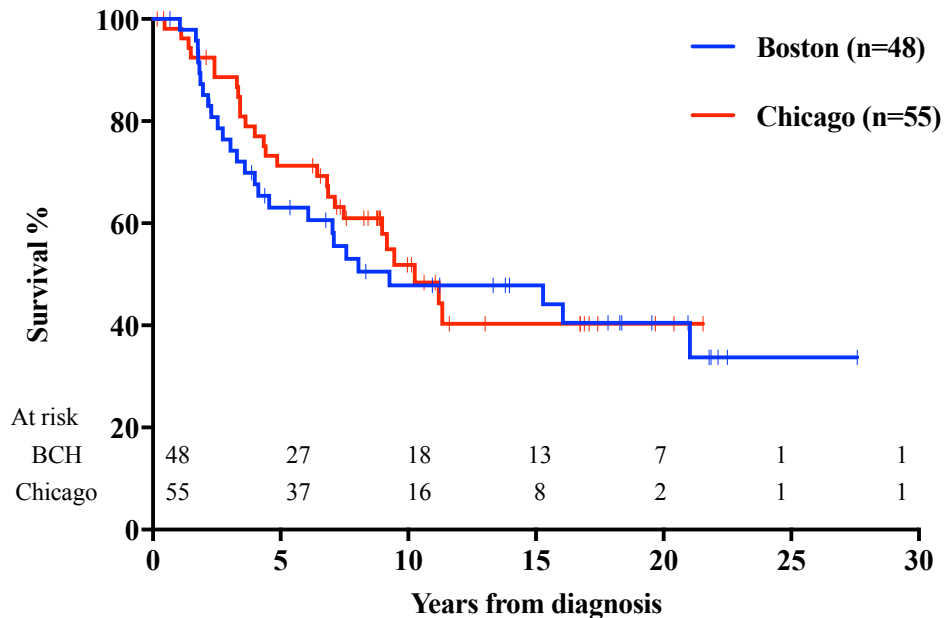

Supplement: Supplementary file 1 — Kaplan–Meier curves of overall survival (OS) and progression-free survival (PFS) by institution. Supplementary material 1 (PDF 41 KB) [file 11060_2017_2568_MOESM1_ESM.pdf]
