# Supplementary material for: Rethinking childhood ependymoma: a retrospective, multi-center analysis reveals poor long-term overall survival
Source: J Neurooncol. 2017 Jul 21;135(1):201–11. doi: 10.1007/s11060-017-2568-8 (PMC5658456; doi:10.1007/s11060-017-2568-8)
Supplement: Supplementary file 2 — Supplementary material 2 (PDF 86 KB) [file 11060_2017_2568_MOESM2_ESM.pdf]

Supplemental Table 1: Patient demographic and clinical characteristics for SEER cohort (n=360)

**Demographic and clinical characteristics**		**Frequency (%)**
Age (years)	<1	30 (8)
	1-4	149 (41)
	5-9	84 (23)
	10-14	51 (13)
	15-19	46 (13)
Gender	Male	206 (57)
Tumor grade	II	82 (23)
	III	278 (77)
Adjuvant XRT	Yes	241 (67)
	No	112 (31)
	Unknown	7 (2)

Supplemental Table 2: Histopathologic features of ependymomas at DFCI/BCH (n=48)

**Histopathologic feature**		**Frequency (%)**
**Architecture**	Well differentiated	13 (27)
	Poorly differentiated	23 (48)
	Dedifferentiated	12 (25)
**Necrosis**	Yes	37 (77)
**Vascular proliferation**	Yes	29 (60)
**P53 status**	Positive	28 (58)
**Blc-2 status**	Positive	42 (88)
**MIB-1 LI**	≥20.5	17 (35)
**Topo-II alpha expression**	≥9.4	14 (29)
**Mitotic index**	>10	8 (17)
**Cyclin D expression**	Positive	36 (75)
